# Elevational and seasonal patterns of plant pollinator networks in two highland tropical ecosystems in Costa Rica

**DOI:** 10.1371/journal.pone.0295258

**Published:** 2024-01-11

**Authors:** E. Jacob Cristóbal-Perez, Gilbert Barrantes, Alfredo Cascante-Marín, Paul Hanson, Beatriz Picado, Nicole Gamboa-Barrantes, Geovanna Rojas-Malavasi, Manuel A. Zumbado, Ruth Madrigal-Brenes, Silvana Martén-Rodríguez, Mauricio Quesada, Eric J. Fuchs

**Affiliations:** 1 Centro de Investigación en Biodiversidad y Ecología Tropical, Universidad de Costa Rica, San José, Costa Rica; 2 Laboratorio Nacional de Análisis y Síntesis Ecológica, Escuela Nacional de Estudios Superiores Unidad Morelia, Universidad Nacional Autónoma de México, Morelia, Michoacán, México; 3 Laboratorio Binacional de Análisis y Síntesis Ecológica, UNAM-UCR, México, Costa Rica; 4 Escuela de Biología, Universidad de Costa Rica, San José, Costa Rica; 5 Investigador Colaborador, Museo de Zoología, Universidad de Costa Rica, San José, Costa Rica; 6 Laboratorio de Ecología Evolutiva de Plantas, Escuela Nacional de Estudios Superiores–Morelia, Universidad Nacional Autónoma de México, Morelia, Michoacán, México; Universidade Federal de Uberlandia - Campus Umuarama, BRAZIL

## Abstract

Many plant species in high montane ecosystems rely on animal pollination for sexual reproduction, however, our understanding of plant-pollinator interactions in tropical montane habitats is still limited. We compared species diversity and composition of blooming plants and floral visitors, and the structure of plant-floral visitor networks between the Montane Forest and Paramo ecosystems in Costa Rica. We also studied the influence of seasonality on species composition and interaction structure. Given the severe climatic conditions experienced by organisms in habitats above treeline, we expected lower plant and insect richness, as well as less specialized and smaller pollination networks in the Paramo than in Montane Forest where climatic conditions are milder and understory plants are better protected. Accordingly, we found that blooming plants and floral visitor species richness was higher in the Montane Forest than in the Paramo, and in both ecosystems species richness of blooming plants and floral visitors was higher in the rainy season than in the dry season. Interaction networks in the Paramo were smaller and more nested, with lower levels of specialization and modularity than those in the Montane Forest, but there were no seasonal differences within either ecosystem. Beta diversity analyses indicate that differences between ecosystems are likely explained by species turnover, whereas within the Montane Forest differences between seasons are more likely explained by the rewiring of interactions. Results indicate that the decrease in species diversity with elevation affects network structure, increasing nestedness and reducing specialization and modularity.

## 1. Introduction

Mutualistic interactions are crucial for maintaining ecological processes within ecosystems [1,2]. For example, plant-pollinator interactions are critical for the sexual reproduction of more than 90% of terrestrial seed plants, as well as the maintenance of genetic diversity, species richness, animal survival, and ecosystem function [3–8]. The effectiveness of animal pollination depends on plant reproductive traits, such as floral morphology and flowering phenology, that have evolved in concordance with the morphology, behavior, and phenology of pollinators [9], which are further shaped by long-term patterns and short-term fluctuations in environmental conditions [10]. Consequently, seasonal and spatial variations in the composition of flowering plants and floral visitor communities modify the structure and dynamics of plant-pollinator interactions [11,12].

Variation in abiotic factors, such as atmospheric pressure, air temperature, and solar radiation, has an impact on the composition and population dynamics of plant and animal communities along elevational gradients [13]. These factors have defined clear ecotones between ecosystems at high elevations [14,15]. In the neotropics, Paramo ecosystems and adjacent Montane Forests converge in a well-defined ecotone, as plant species composition and vegetation structure differ significantly along a short elevational gradient [15,16]. The Paramo is dominated by an herbaceous stratum interspersed with shrub-thickets, in stark contrast to the arboreal vegetation that characterizes the adjacent Montane Forests. Blooming plant richness in the Paramo has been shown to be consistently lower than in the Montane Forest [16]. Studies have shown that differences in the availability of floral resources (i.e., nectar and pollen) between habitat types are important determinants of plant-visitor interactions [17]. However, information on floral-visitor assemblages and their interactions with plants in high-elevation tropical ecosystems is scarce [18–22].

High-elevation tropical ecosystems stand out as regions of high endemism but also as emerging climate change hot-spots [23]. Climate models predict that these ecosystems will experience a strong decrease in precipitation, higher temperatures, and an increase in the variability of both precipitation and temperature [24]. While previous work has demonstrated that changes in climatic conditions in high-elevation tropical ecosystems can have an impact on biodiversity [25,26], we currently lack a comprehensive understanding of the potential implications of these changes on mutualistic interactions [20]. Changes in abiotic conditions due to climate change are expected to cause a reduction in population sizes and species distribution ranges, which may lead to local extinctions [27–29]. Phenological mismatches between flowering plants and their pollinators have also been documented in highland ecosystems as a consequence of climate change in temperate regions [30–34], but this phenomenon has scarcely been studied in tropical regions. Therefore, understanding how species interactions are structured across tropical high montane environments would provide information on climate factors affecting species vulnerability and community structure in these endangered habitats.

Ecological communities are dynamic [35], particularly in seasonal ecosystems where phenological patterns of plants and their pollinators often induce large, temporal changes in the diversity and composition of floral resources and animal pollinators [10,12,36]. This temporal variation in floral resources may constrain plant–pollinator interactions at certain times of the year [37–39]. In a recent study conducted in the Paramo and Montane Forest ecosystems of Costa Rica, the flowering peak of insect-visited flowers occurred in the rainy season, while the flowering peak of bird-visited flowers occurred in the dry season [16]. As a result of seasonal changes in flowering phenology and animal species composition, the occurrence and intensity of ecological interactions may also vary over time [40]. Changes in plant pollinator interactions over spatial and temporal scales may be attributed to shifts in species assemblages (i.e., species turnover) or their plasticity to interact with other species (i.e., interaction rewiring) [12,41–44]. While rewiring appears to drive the temporal persistence of interactions at seasonal scales [43,45], species turnover may be more important across a spatial scale [41,46,47]. Only a few studies have simultaneously studied the role of both spatial and seasonal variation in the structuring of plant-pollinator networks [12,43,48–50]. Studying the drivers that influence interaction networks in highland ecosystems should improve our understanding of these highly endangered ecological communities.

In this study, we analyzed the spatio-temporal variation in plant-pollinator interaction dynamics in two highland tropical ecosystems in Costa Rica (Paramo and Montane Forest) over a period of two years. To assess how variation in floral resource availability may influence the community of floral visitors, we contrasted patterns of abundance and diversity of floral visitors (insects and hummingbirds) between the two ecosystems. We also reconstructed interaction networks and evaluated how the structure and beta diversity of these networks differ between ecosystems and seasons. Since abiotic conditions become more stressful as elevation increases and a reduced number of species are able to establish and survive, plant and floral visitor diversity are expected to decrease at higher elevations [51–57]. Consequently, we anticipate a reduction in the size of plant-visitor networks as elevation increases. Contrary to Olesen and Jordano´s proposal of greater specialization in higher elevation communities [58], we expect less specialization in the Paramo, based on the ecological optimality hypothesis [59–61]. This hypothesis predicts that defense, and hence resource competition, is strong when benefits are also high, and that this is likely to occur within a specific range of the resource abundance distribution, limited by a lower and higher threshold. When resources are limited, the costs of defending them outweigh the benefits. When resources are scarce (below the lower-level threshold), the cost of defending them may outweigh the benefit obtained from them, particularly for territorial animals (e.g., some hummingbird species). On the contrary, if resources exceed the upper-level threshold, competing and non-competing individuals are expected to obtain similar benefits [59–61]. As a result, in the Paramo, where floral resources and activity hours are limited (e.g., due to colder temperatures and severe winds), competition for floral rewards should be relaxed to allow for the quickest possible resource acquisition. This would result in greater resource sharing and less specialization in plant-floral visitor networks. The rainy season is expected to have a greater diversity of blooming plants and floral visitors, as well as larger networks, because short-lived plants are more likely to flower in this season [16,62].

## 2. Methods

### 2.1 Study area

This study was conducted at two adjacent sites in Cerro de la Muerte, Talamanca Mountain Range (09° 33’ N; 83° 44’ W), Costa Rica. The two study sites, Quetzales National Park (QNP) and Cerro de la Muerte Biological Station (CMBS), are located at 3500 m asl (Paramo) and 3000 m (Montane Forest) respectively. Temperature oscillates drastically during the day, particularly in the Paramo (-5° to 35°; mean 7.6°C), whereas the mean temperature in the CMBS is 11°C (6° min. - 22° max.) [63,64]. The mean annual precipitation in the region is 2500 mm, with a relatively dry period from November to April [63]. In the Paramo, the vegetation is dominated by an herbaceous stratum with a large diversity of Asteraceae and Poaceae and scattered patches of shrubs with species mainly in the Ericaceae, Asteraceae, and Hypericaceae [65]. The vegetation in the adjacent Montane Forests is dominated by oaks (*Quercus costaricensis* Liebm.) with abundant epiphytes and shrubs and many species in the Ericaceae, Asteraceae, and Onagraceae [66].

### 2.2 Data collection

We established two 10 m by 2 km long transects separated by 1 km at each site. During a 20-month period (September 2020 to April 2022), we conducted monthly surveys of all blooming plant species and collected floral visitors. During each survey, we walked at a steady pace, registering all flowering plants of each species along each transect; wind-pollinated grasses (Poaceae) and sedges (Cyperaceae) were not included in the study. We collected data along the same transects as a prior study [16], ensuring consistency in the study area and methods. Because we surveyed blooming plants and conducted the experiments at different times, the species included in this study differ from our earlier findings. Simultaneously, we collected insect floral visitors from plants of nearly all blooming species using entomological nets and plastic bags. All insects collected in the field were deposited in 80% ethanol and labeled for later identification. We also recorded hummingbird and bumblebee (*Bombus ephippiatus*) visits through direct observation of flowers. Each transect was surveyed by at least two observers from 8:00 to 12:00 h. We collected samples from floral visitors throughout the day for the initial two to three weeks. Nevertheless, afternoons in PA and MF are often cloudy and rainy, and the temperature decreases rapidly, drastically reducing insect activity. To maximize human resources, we opted to sample insects exclusively in the morning, when insect and hummingbird activity is highest. We are aware that we might have missed some floral visitors that occasionally forage in the afternoon, but considering the low to nearly null insect activity during this time, we consider that the missing samples did not affect the results obtained in our research. Insects collected on flowers were identified to the lowest possible taxonomic level using the relevant keys [67–69] and compared with identified specimens in the entomological collections of the Museo de Zoología, Escuela de Biología, Universidad de Costa Rica, and Museo Nacional de Costa Rica. Voucher specimens of all collected insects are deposited in the Museo de Zoología, Escuela de Biología, Universidad de Costa Rica.

### 2.3 Data analysis

#### 2.3.1 Species diversity of flowering plants and floral visitors

We calculated the effective number of species using ^q^D (*sensu* Jost [70]) to estimate the diversity of blooming plant species and floral visitors in the two ecosystems (i.e., Paramo and Montane Forest). We calculated three metrics of diversity: (1) zero-order (^0^D), which is equivalent to species richness; (2) order 1 (^1^D), where all species are weighted proportionally to their abundance in the assemblage; and (3) order 2 (^2^D), which gives more weight to dominant species in the assemblage, as implemented by Cortes et al. [12]. We performed a Kruskal Wallis test to evaluate differences in species diversity (dependent variable) between ecosystems. We used the Bray-Curtis index (BC) [71] to evaluate the similarity of plant species and floral visitors across ecosystems and seasons. Similarity was also visualized using a non-metric multidimensional scaling (NMSD) based on a Bray-Curtis dissimilarity matrix with 1000 permutations. We performed a distance based Permutational Multivariate Analysis of Variance (PERMANOVA) using the *adonis* function from the vegan R package [72] to test for differences in community composition between sites (Montane Forest and Paramo) and seasons (dry and rainy seasons). Diversity analyses were performed using the ‘vegan’ [72], ‘BiodiversityR’ [73], and ‘iNEXT’ [74] packages in R [75].

#### 2.3.2 Interaction networks

We constructed and compared interaction networks for each season (dry and rainy) in each ecosystem (MFD = Montane Forest Dry; MFR = Montane Forest Rainy; PD = Paramo Dry; PR = Paramo Rainy). We used various network metrics: (1) the H2’ specialization index, which ranges from 0 to 1 with increasing specialization [76,77]; (2) the nestedness value using NODF (Nestedness based on Overlap and Decreasing Fill) and weighted NODF (WNODF), metrics that measure the extent to which interactions of specialized species are subsets of interactions of more generalist species in the network, ranging from 0 (not nested) to 100 (perfectly nested); (3) the connectance, a metric that calculates the observed number of interactions divided by the total number of possible interactions in a network [78]; (4) the weighted mean number of interacting partners which estimates pollinator generality and plant vulnerability [79,80]; (5) the Q modularity test to determine whether groups of species within networks interact more strongly than expected by chance based on a null-model (*r2d* algorithm) [81]; and (6) interaction evenness which compares the homogeneity between interaction frequencies [79]. We compared each network metric with a null model following Dormann et al. [82], to determine whether network metrics are the result of random interactions. The null model is based on 1000 permutations, maintaining the matrix’s row/column sums constant. For this analysis, we used the *r2dtable* null model function in the ‘*bipartite*’ R package [83]. Network metrics were deemed significant when empirical metrics were larger than 95% relative to the metrics from randomly generated networks.

To identify the role of each species within a modular network, we computed the degree to which each species is connected to other species within their module (i.e., within-module connectivity, *z*) and the degree to which interactions of a given species are distributed across modules (among-module connectivity, *c*) [84,85]. Species can then be classified as peripherals (i.e., specialist species with few interactions with other species), connectors (i.e., generalist species connecting several modules to each other), module hubs (i.e., generalist species with many interactions only within their own modules), or network hubs (i.e., generalist species that have both many interactions within their own modules but also connect several modules to each other). We standardized the network metrics to standard normal deviations (*z*-scores) to compare the network structure values between the studied ecosystems and seasons. A z-score below 1.65 indicates that the analyzed metrics fall below 95% of the empirical distribution obtained from the null model (i.e., P > 0.05) [86]. The reference values for the metrics follow Olesen et al. [84].

#### 2.3.3 Dissimilarity of species interaction networks

We estimated the dissimilarity of species interaction networks [42] (Poisot et al., 2012) as the turnover of plant-floral visitor interactions using the Whittaker dissimilarity index estimated by the *’betalinkr’* function in the ’bipartite’ R package. This index estimates the extent to which specific plant-floral visitor interactions differ between ecosystems and seasons. We compared networks between seasons for each ecosystem, and between ecosystems within a season. With ‘*betalinkr*’ we calculated two additive components of beta diversity (β_WN_): composition turnover (β_ST_) and interaction rewiring (β_OS_) in the species shared between seasons and ecosystems. Additionally, we calculated the relative contribution of compositional differences given by (βST/βWN) × 100. This index ranges between 0 and 100%, where values of βST/βWN > 50% suggest a high turnover, reflecting large differences in the species composition and interactions between networks. Values of βST/βWN < 50% indicate a low turnover of species and a greater influence of species rewiring on beta diversity. For all comparisons, we used the ‘commondenom’ method to analyze β diversity components between networks. We used the ‘bipartite’ package [83] in the R statistical language [75] to compute all the network metrics.

## 3. Results

### 3.1 Blooming plant and floral visitor species diversity

We recorded 71 blooming plant species in 61 genera and 32 families, 59 species in the Montane Forest and 46 in the Paramo. Asteraceae was the most diverse family, with 13 species in each ecosystem. The three parameters of diversity estimated for blooming plant species richness (^0^D), proportional abundance (^1^D), and dominant species (^2^D) were significantly higher in the Montane Forest than in the Paramo (H _1,12_ = 6.08, p = 0.016; H _1,12_ = 9.81, p = 0.001; H _1,12_ = 4.85, p = 0.027; Table 1).

**Table 1:** Diversity metrics of plants and floral visitors in two highland Costa Rican ecosystems.

Group	Ecosystem	^0^D	^1^D	^2^D
Plants	Montane Forest	66 (2.26)	35.92(0.93)	24.44(0.93)
	Paramo	47 (1.76)	29.70 (0.79)	23.64 (0.83)
Floral visitors	Montane Forest	77 (20.54)	14.36 (0.49)	5.83 (0.24)
	Paramo	73 (37.21)	9.76 (0.44)	4.71(0.17)
			

Species richness (^0^D), and the effective number of species, considering all species proportionally weighted according to their abundance (^1^D), and weighting the most abundant species (^2^D). Standard error in parentheses.

We collected 3968 floral visitors, which we separated into 100 morphospecies belonging to 58 genera in 55 families. Diptera was the most species rich group (72 spp., with predominance of Muscidae and Syrphidae. Flies were followed by bees (4 spp.), with *B*. *ephippiatus* (Apidae) as the most common species in both habitats, and hummingbirds (4 spp.), of which only one species (*Selasphorus flammula*) was present in both ecosystems. Other insect groups such as beetles (11 spp.), wasps (4 spp.), butterflies (2 spp.), and Hemiptera (1 sp.) had fewer species. We registered 77 species of floral visitors in the Montane Forest and 73 in Paramo. The species richness (^0^D) of floral visitors did not differ between ecosystems (H _1,12_ = 3.44, p = 0.06; Table 1). However, the effective number of floral visitors was greater in the Montane Forest, when we considered the proportional abundance (^1^D) (H _1,12_ = 6.45, p = 0.011; Table 1), and weighted dominant species (^2^D) (H _1,12_ = 5.07, p = 0.024; Table 1).

The multidimensional scaling distances showed that the composition of blooming plant species differed between ecosystems and seasons, and the interaction of both factors was also statistically significant (F_1,12_ = 48.56, p = 0.001; F_1,12_ = 15.72, p = 0.001; F_1,12_ = 9.74, p = 0.001, respectively) (S1A Fig). The composition of floral visitors also differed between ecosystems and seasons, but the interaction was not significant (F_1,12_ = 6.81, p = 0.001; _F1,12_ = 3.37, p = 0.002; F_1,12_ = 0.92, p = 0.49, respectively) (S1B Fig). We found that the Montane Forest and the Paramo shared 43 species of blooming plants and 50 species of floral visitors. Dissimilarity indices indicated that the composition of blooming plants and floral visitors differed between the two ecosystems (S1 and S2 Tables). Additionally, species composition differed between dry and rainy seasons, and these differences were more pronounced for blooming plant communities (S1A and S1B Fig) (S1 and S2 Tables).

### 3.2 Plant-floral visitor interaction networks

We found that plant-floral visitor networks differed in structure between Montane Forest and Paramo and between seasons (Table 2). Network size, number of links, and network weight were higher in the Montane Forest than in the Paramo. Networks were larger in size and weight during the dry season in the Montane Forest, but showed the opposite pattern in the Paramo, with larger networks during the rainy season (Table 2). All networks were nested (NODF, WNODF parameters), with slightly higher values in the Montane Forest than in the Paramo, but differences were not significant between the dry and rainy seasons at either site (Table 2; S1 Table). Modularity and species specialization of plants and flower visitors were also higher in the Montane Forest than in the Paramo (S2 Fig). Both metrics increased during the rainy season in both ecosystems, but the specialization of floral visitors was similar between seasons (Table 2; S3 Table). Interaction evenness and connectance did not differ between habitats or seasons (Table 2; S3 Table).

**Table 2:** Ecological network metrics for rainy and dry seasons in Paramo and Montane Forest in Costa Rican highlands.

	Montane Forest		Paramo	
Metrics	Rainy	Dry	Rainy	Dry
Size	100	108	88	86
Visitor species	57	61	51	52
Plant species	43	47	37	34
Links	215	259	186	180
Weight	1046	1203	906	813
Nestedness (NODF)	**27.99**	**30.14**	**34.99**	**33.01**
Weighted nestedness (WNODF)	**16.26**	**15.22**	**22.64**	**17.02**
Modularity (*Q*)	**0.55**	**0.41**	**0.39**	**0.30**
*H2’*	**0.49**	**0.41**	**0.33**	**0.34**
Connectance	**0.09**	**0.08**	**0.09**	**0.10**
Interaction evenness	**0.55**	**0.58**	**0.58**	**0.55**
Generality (visitors)	**7.12**	**10.8**	**12.09**	**8.46**
Vulnerability (plants)	**4.35**	**4.73**	**4.25**	**4.70**

Estimated network metrics were compared with random-generated models (N = 1000). Bold values indicate a significant difference from random expectations (p < 0.01).

Species play different roles in networks based on their within-module *z* and among-module connectivity *c*. In this study, a high proportion of species were peripheral (i.e., specialists with low z- and *c*-values) across networks, with most of their links found within their modules (MFD = 78%, MFR = 87%, PD = 73%, PR = 74%). A large fraction (MFD = 52%, MFR = 43%, PD = 46%, PR = 66%) of these specialist species had *c*-values = 0, indicating that their module had no external links (S3 Fig). During the dry season, 22% of the species in the Montane Forest were generalists (20% connectors with low *z* and high *c*-values, and 2% network hubs with high *z* and low *c*-values), whereas 13% of the species were generalists (all them connectors) during the rainy season (S3 Fig). In the Paramo, during the dry season, 27% of the species were generalists (25% connectors and 2% network hubs), and during the rainy season, 26% were generalists (22% connectors and 4% network hubs) (S3 Fig). We did not find species functioning as module hubs (with high *z* and low *c*-values) in any of the networks (S3 Fig). Seven fly species (Muscidae, Syrphidae, Ephydridae), two bee species (Andrenidae: *Protandrena* sp., and Halictidae: *Lasioglossum* sp.), and one wasp species (Vespidae: *Polybia aequatorialis*) were the most common connectors in Montane Forest. In the Paramo, the connectors were nine fly species (Muscidae, Syrphidae, Anthomyiidae, Simuliidae), four bee species (*Bombus ephippiatus*, *Apis mellifera*, *Protandrena* sp., *Lasioglossum* sp.) and one beetle species (Curculionidae).

Networks differed between seasons within each ecosystem (β_WN_ > 0.70). In the Montane Forest, differences between seasons were better explained by a rewiring of interactions than by species turnover (β_ST_ / β_WN_ < 50%) (Table 3). In contrast, in the Paramo seasonal differences were explained by species turnover (β_ST_ / β_WN_ > 50%) (Table 3). Similarly, comparing interaction networks between ecosystems during the same seasons (MFD vs. PD; MFR vs. PR) revealed that differences in network structure can be attributed to species turnover (Table 3).

**Table 3:** Beta diversity between interaction networks (β_WN_) is composed of the dissimilarity in species composition (β_ST_) and the dissimilarity in shared species interactions (β_OS_).

Network-1	Network-2	β_ST_	β_OS_	β_WN_	β_ST_/β_WN_
Montane Forest D	Montane Forest R	0.30	0.42	0.73	0.41
Montane Forest D	Paramo D	0.56	0.24	0.80	0.69
Montane Forest D	Paramo R	0.62	0.24	0.86	0.72
Montane Forest R	Paramo D	0.58	0.21	0.80	0.72
Montane Forest R	Paramo R	0.42	0.33	0.76	0.55
Paramo D	Paramo R	0.40	0.30	0.70	0.56

β_ST_/β_WN_ values express the relative contribution of the compositional and interactive components to Beta diversity. D: Dry season, R: Rainy season.

## 4. Discussion

**Fig 1:**
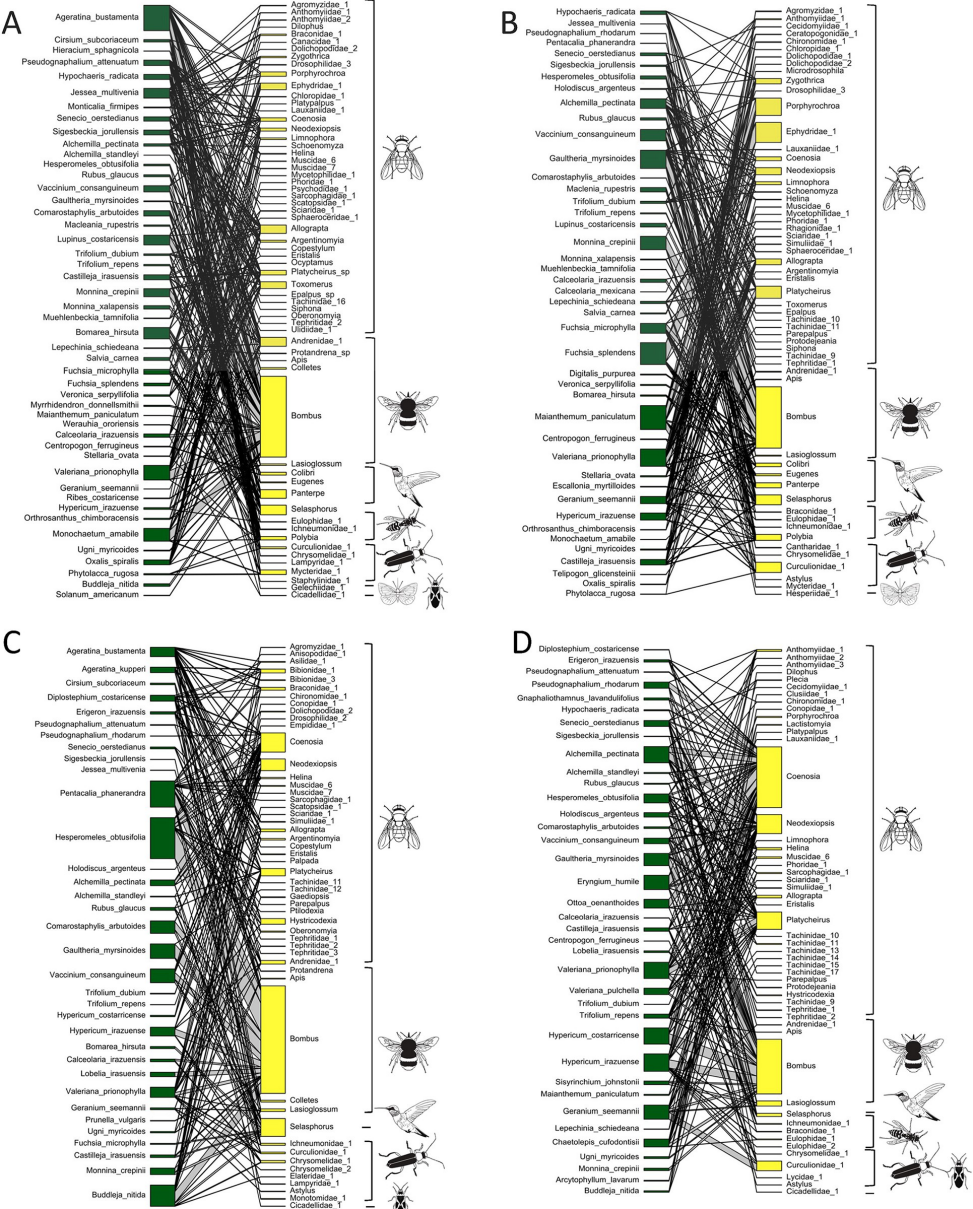
Plant-floral visitor networks in the rainy and dry seasons in the Montane Forest and Paramo. Green boxes represent plant species and yellow boxes floral visitors identified to the lowest possible taxonomic level. Icons represent floral visitor groups. A: Montane Forest Dry, B: Montane Forest Rainy, C: Paramo Dry, D: Paramo Rainy.

Spatio-temporal variation in floral resources is expected to influence plant-pollinator interactions [37–39]. In this study, we found that diversity of blooming plant species and floral visitors was lower in the Paramo than in the Montane Forest. Although the studied ecosystems are separated by only a short elevational range (500 m), this difference plays a determining role in species composition and diversity [16]. Several historical and ecological factors have likely acted synergistically to produce the observed differences in diversity [87–91]. Differences in plant species composition are likely related to biogeographical events, such as plant dispersal events that occurred during the late Pleistocene [91,92], the subsequent geographic isolation, and the prevalence of cold, seasonal climatic conditions in the Paramo, in contrast with the milder climate (i.e., warmer, misty, and less seasonal) prevailing in the Montane Forest [93]. This has resulted in a unique Paramo vegetation that differs notably from the adjacent Montane Forest [16]. Similarly, climatic conditions in the Paramo may also explain the lower diversity of floral visitors [94]. The large daily fluctuation in temperature (from below freezing to >30°C) may physiologically constrain the development of different insect species, reducing the diversity of insects to those capable of tolerating such temperature variation [95]. Availability of resources is another factor that affects species diversity [96]. Low energy demanding groups, such as flies, are more abundant at high elevations and latitudes, in contrast with bees and other high energy demanding groups, such as hummingbirds [17,21,97,98]. In our study, we recorded four hummingbird species in the Montane Forest and only one species (the smallest hummingbird at this elevation) in the Paramo (Fig 1; [99]). Although some rare floral visitors may have been missed by sampling during periods of high activity in the morning.

Seasonal differences in the blooming plant communities and floral visitors found in our study are likely a response of different plant and floral visitor groups to environmental cues and resource availability [96,100–104]. In seasonal ecosystems, flowering patterns in some groups of plants are related to differences in water acquisition and resource storage strategies [62], whereas the phenology of floral visitors generally correlates with plant flowering phenology in response to resource availability [105]. For instance, the flowering of most annual herbaceous species occurs during the rainy season, which are mainly visited by insect species, as has been documented in Cristóbal-Pérez et al., [16]; whereas shrubs (*Macleania rupestris*, *Gaultheria myrsinoides*, *Vaccinium consanguineum*) and epiphytic perennials (*Werauhia ororiensis*, *Bomarea hirsuta*) extend their flowering phenology between seasons and are mostly visited by hummingbirds and bumblebees [16,106]. This is consistent with results obtained in other seasonal tropical ecosystems, where insect species that visit flowers are more abundant during the rainy season, while hummingbirds are common visitors year-round [50,107]. In addition to changes in species composition, our results show changes in the frequency of floral visits in some species as a likely consequence of seasonal fluctuations in population size or foraging activity. For instance, the fly genera *Platycheirus* (Syrphidae), *Porphyrochroa* (Empididae), *Coenosia* and *Neodexiopsis* (Muscidae), are more frequent in the rainy season than in the dry season, possibly due to an increase in the availability of floral resources (Fig 1).

In both ecosystems and seasons, interaction networks show a nested structure, which results from asymmetrical interactions. Nestedness occurs when a set of specialized plant species are visited by a small subset of floral visitors, which also visit more generalized plant species; meanwhile, specialized floral visitors use a subset of the more generalist blooming plant species [108]. In our study, we documented a high proportion of specialist insect species foraging at plants that are visited by a wide spectrum of floral visitor species and a lower proportion of generalist insect species foraging at specialist and generalist plants alike (Figs 1 and S3). For example, several flies (Diptera) (almost 50% of the species) interact with only one generalist plant species (S3 Fig), but other flies (e.g., species of *Coenosia*, *Platycheirus*, *Neodexiopsis*, *Allograpta* and *Helina*), are abundant year-round and maintain a large connectivity among and within modules in both ecosystems (S3 Fig). Unlike other studies [84,109], the modules identified in the networks did not represent pollination syndromes. The lack of an association between modules and pollination syndromes could be related to the fact that we recorded floral visitors instead of effective pollinators. Nonetheless, the relationship between modules and pollination syndromes has been found in studies that have determined pollinator effectiveness [109] and others that have not (e.g., [84]). Therefore, the lack of an association between modules and syndromes may be related to the presence of super-generalist pollinators in high-elevation environments [110,111]. The presence of these super-generalist floral visitors in all modules may disrupt the signature of floral syndromes [110].

The interaction networks in the Montane Forest had a higher degree of specialization and modularity than those in the Paramo. This may be caused by factors such as the availability of floral resources and the ecological relationships between plants and their pollinators [112]. According to optimal foraging theory, organisms have evolved to maximize their fitness by optimizing traits and behaviors to adapt to their environment [59,60]. The hypothesis recognizes that resources are frequently limited and that organisms must make trade-offs when allocating resources to various tasks. For example, organisms optimize their foraging strategies by deciding what to consume to maximize calorie intake while minimizing energy expenditure. When resources are scarce, competing for them is untenable since the costs exceed the benefits of defending them. Therefore, organisms tend toward generalization to obtain energy in the shortest possible time [50,113–115]. Highly specialized pollinators may struggle to discover enough of their favorite flowers and may have decreased foraging success. As predicted, a combination of blooming species with fewer resources (e.g., lower nectar volume) and fewer daily foraging hours (e.g., because of extreme abiotic conditions) in the paramo, encourages resource acquisition from multiple floral resources. During the dry season, when fewer plant species are in bloom, *Coenosia* flies (Muscidae) visited up to four times as many species in the Paramo compared to Montane Forest (81% vs. 17%). Similarly, the hummingbird *Selasphorus flammula* visits up to 29% of the flowering species in Paramo, compared with 23% in the Montane Forest. In addition, plant species almost exclusively visited by hummingbirds (e.g., *B*. *hirsuta*, *W*. *ororiensis*, *M*. *rupestris*, *Centropogon ferrugineus*, *Cirsium subcoriaceum*), are more common in the Montane Forest than in Paramo. This would increase specialization in the more diverse ecosystem at lower elevations, which is in direct contrast with previous expectations that specialization decreases with elevation and lower diversity [50]. Our findings suggest that resource limitation is more likely to influence generalization in interaction networks in the Paramo than in the Montane Forest.

As previously discussed, interaction dissimilarity was high between networks in different seasons within ecosystems. In the Montane Forest, interaction dissimilarity between seasons is related to interaction rewiring, where co-occurring species interact differently in each season. On the contrary, in the Paramo, dissimilarity between seasons is primarily caused by species turnover. Some generalists and abundant visitors, such as *Bombus*, hummingbirds, and some flies, are present year-round and their interactions with blooming plants are rearranged between seasons, which could explain species rewiring observed in the Montane Forest. Network theory predicts that rewiring occurs when resources (e.g., blooming species) are lost from the community [116,117]. However, if blooming intensity increases in one or a few plant species, these may attract more floral visitors, changing their interactions temporarily [118] and thus rewiring the interactions of flower visitors in the community without species turnover. Interaction rewiring indicates the high flexibility of both floral visitors and plants in adjusting interactions to distinct ecological scenarios, such as variations in floral resource diversity and abundance [12]. This flexibility is an important ecological trait in these ecosystems that could make them more resilient to perturbation or species extinction; networks with a higher proportion of generalists and a greater capacity for rewiring are thought to be less likely to collapse [116]. In contrast, the observed interaction turnover between seasons in the Paramo is more likely caused by a higher seasonal dissimilarity in blooming plant and floral visitor communities relative to the Montane Forest. The harsh conditions in the Paramo, where flowering plants have evolved to flower under severe climatic conditions, may explain this finding. The Paramo exhibits large differences in weather patterns between the dry and rainy seasons, which are more pronounced than those in the Montane Forest [63]. The presence of a dry season characterized by low temperatures and severe water deficit may constrain the populations of floral visitors and plant species that survive in the Paramo ecosystem. During the rainy season, heavy rains and short periods of direct solar radiation likely influence flowering phenology and pollinator abundance, resulting in significant differences in seasonal species assemblages [16]. For example, wind-dispersed species must flower during the dry season to disperse fruits before the heavy rains begin, and this is particularly important for species of Asteraceae. Additionally, morning temperatures in the Paramo can drop below freezing from December through February (dry season) [63], which may be demanding for high energy pollinators such as large bees, large flies, and hummingbirds. These climatic filters likely shape plant-pollinator interactions, resulting in a high seasonal turnover in the Paramo.

We found that floral visitors within the interaction networks show high levels of specialization. Highly specialized networks are more susceptible to habitat perturbation and species extinction. Given that both Montane Forest and Paramo are very susceptible to changes in species abundance and composition due to climate change, this may lead to mismatches between the flowering of plant species and their floral visitors [119]. In our study, several plant species interacted with only one floral visitor (MFD = 21%, MFR = 32%, PD = 29%, PR = 16%, respectively). Most empirical and theoretical studies suggest that nested and asymmetric networks are robust to species loss [84] and changes in phenology [30]. However, these studies also agree that the loss of generalists will have an important impact on network structure and stability. In the Paramo and the Montane Forest, generalist pollinators like *Bombus ephippiatus*, *Allograpta* sp. (Syrphidae), and *Coenosia* sp. (Muscidae), are important connectors/network hubs, but their role as generalists changes seasonally, which suggests that they are susceptible to changes in resource availability and may be adapted to specific climatic conditions (e.g., temperature, precipitation). In the Montane Forest, the number of generalists decreases during the rainy season, which may be a consequence of heavy rains that limit the foraging activity of many insect species.

Phenological changes may decouple the availability of floral resources and floral visitors with possible negative consequences for population sizes of floral visitors and plant reproductive success [120]. Continuous flowering phenology and a more diverse flowering plant community in Montane Forest are likely to provide more constant floral resources year round to maintain viable populations of floral visitors. In contrast, the Paramo habitats adjacent to the Montane Forest occur at higher altitudes, present smaller areas, a more restricted flowering span and more extreme environmental conditions. Under such conditions, Montane Forest could be regarded as a source of floral resources and floral visitors, whereas the adjacent Paramo may function as a sink for floral visitors due to its limited availability of floral resources and extreme environmental conditions [121]. Thus, under scenarios of climate change or land-use change, the conservation of floral visitor populations in the paramo and the sexual reproduction of animal-pollinated Paramo plants may possibly depend on the conservation of the mosaic of populations of adjacent Montane Forests.

## 5. Conclusions

In summary, we found that seasonality, as well as differences between the two ecosystems in richness and beta diversity of blooming plants and floral visitors, determine the structure and dissimilarity of the studied plant-pollinator networks. Our results show that the richness and diversity of blooming plants and floral visitors are lower in the Paramo than in the Montane Forest. Climatic constraints imposed by elevational changes likely explain the reduction in blooming plant diversity in the Paramo, while the synergistic effect of vegetation structure, severe climatic conditions, and the availability of food resources reduce floral visitor richness and diversity. This supports the argument that resource depletion may limit the use of the Paramo for nectar-feeding birds and insects [106,122,123]. Higher species richness and diversity explain the higher specialization and modularity in Montane Forest interaction networks, which is congruent with the argument that increasing the number of species increases interspecific competition, resulting in greater specialization [58]. Interaction turnover between ecosystems was primarily driven by differences in species composition and in the Paramo there were also differences between seasons. This study showed that analysis of species composition and richness based on flowering phenology are useful to evaluate potential floral resources for floral visitors (insects and birds). This study also reveals that comparative analyses between ecosystems and seasons are useful in understanding the spatio-temporal factors that determine the dynamics of plant-pollinator relationships in ecosystems highly threatened by habitat loss and climate change [124].

## Supporting information

S1 Fig Effect of ecosystem and season on the beta diversity of blooming plant species (A) and floral visitors (B), in the Costa Rican Talamanca mountain range. (DOCX)

S2 Fig Modules calculated for each pollination network. (DOCX)

S3 Fig Distribution of floral visitors according to their network role. (DOCX)

S1 Table Bray-Curtis dissimilarity values of blooming plant communities between ecosystems and seasons. (DOCX)

S2 Table Bray-Curtis dissimilarity values of floral visitor communities between ecosystems and seasons. (DOCX)

S3 Table Z-scores of each network metric for each season and ecosystem. (DOCX)
