# Supplementary material for: Elevational and seasonal patterns of plant pollinator networks in two highland tropical ecosystems in Costa Rica
Source: PLoS One. 2024 Jan 11;19(1):e0295258. doi: 10.1371/journal.pone.0295258 (PMC10783733; doi:10.1371/journal.pone.0295258)
Supplement: S1 Fig — (DOCX) [file pone.0295258.s001.docx]

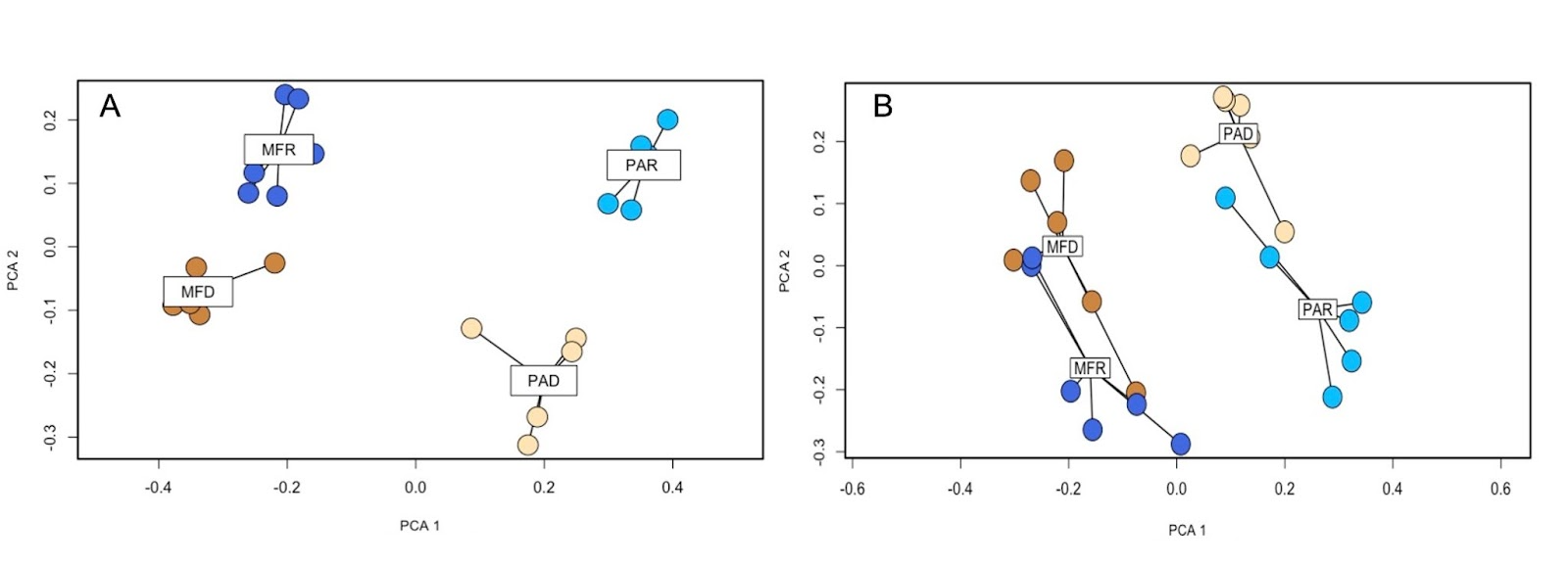


**Fig S1**. Effect of ecosystem and season on the beta diversity of blooming plant species (A) and floral visitors (B), in the Costa Rican Talamanca mountain range. Each dot represents the mean non-Euclidean distance of blooming plants at a particular sampling date relative to the centroid of all samplings on the two first PCA components. MFR: Montane Forest - rainy season; MFD: Montane Forest - dry season; PAR: Paramo - rainy season; PAD: Paramo - dry season.
