# Supplementary material for: Elevational and seasonal patterns of plant pollinator networks in two highland tropical ecosystems in Costa Rica
Source: PLoS One. 2024 Jan 11;19(1):e0295258. doi: 10.1371/journal.pone.0295258 (PMC10783733; doi:10.1371/journal.pone.0295258)
Supplement: S2 Fig — (DOCX) [file pone.0295258.s002.docx]

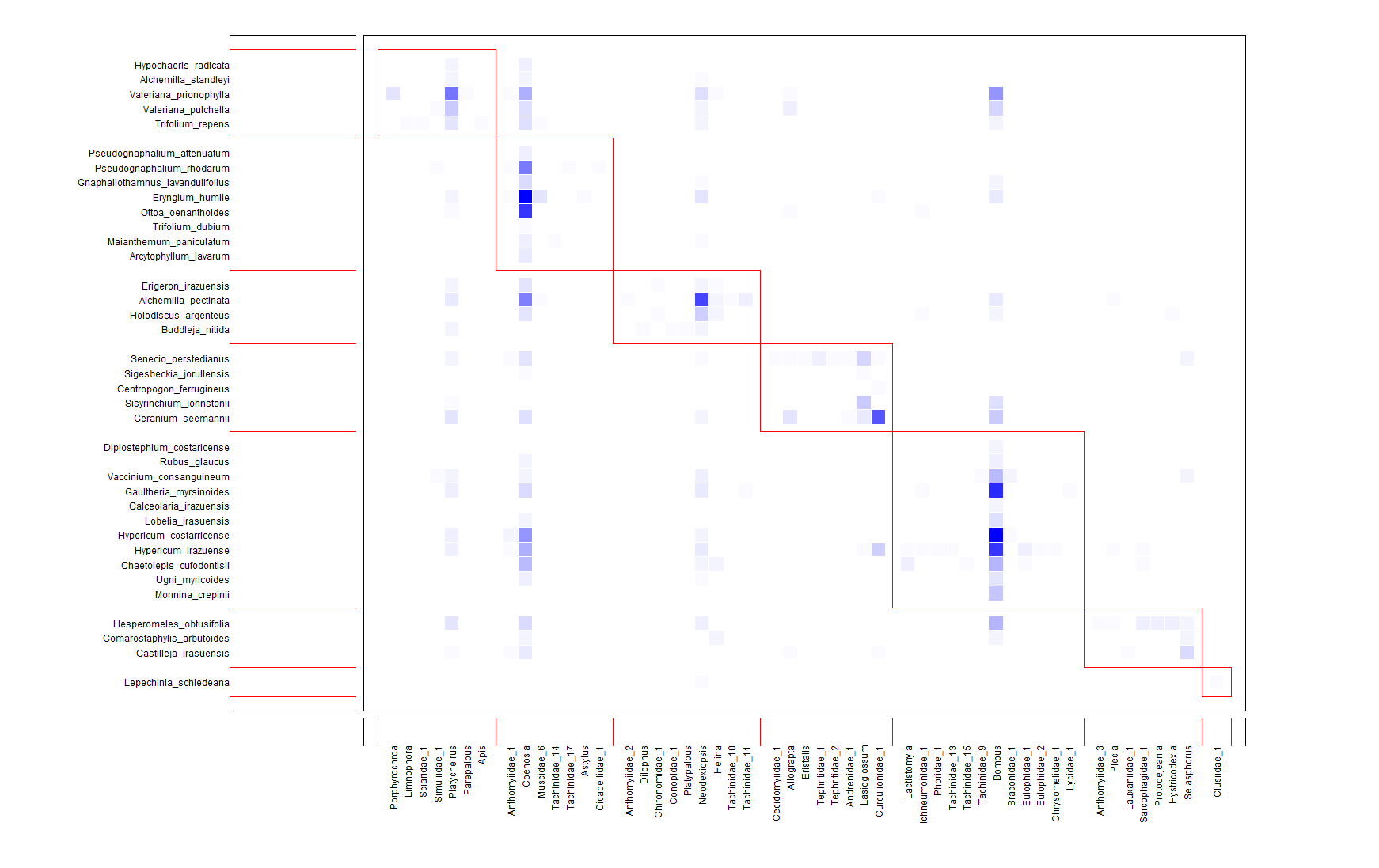

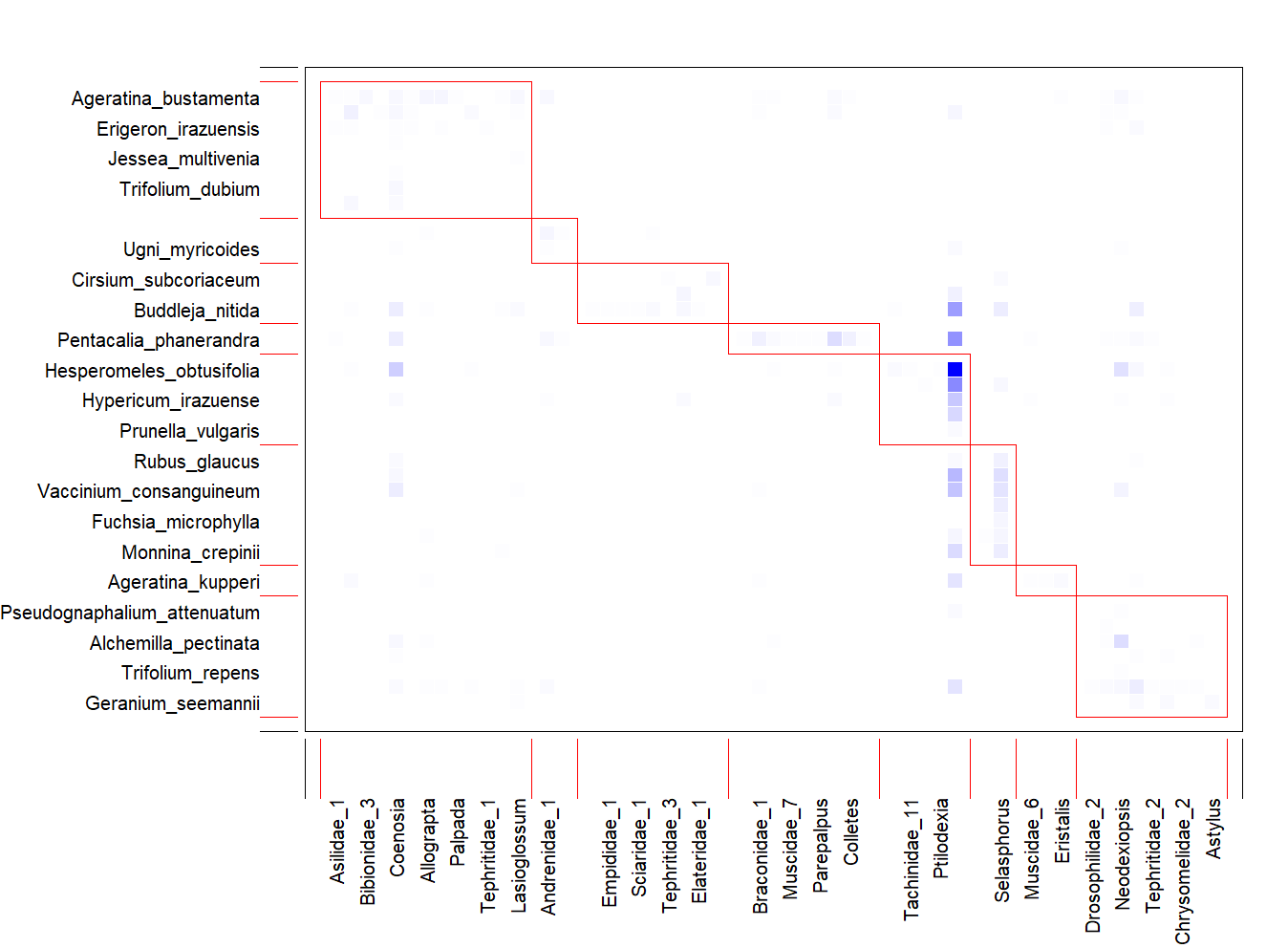

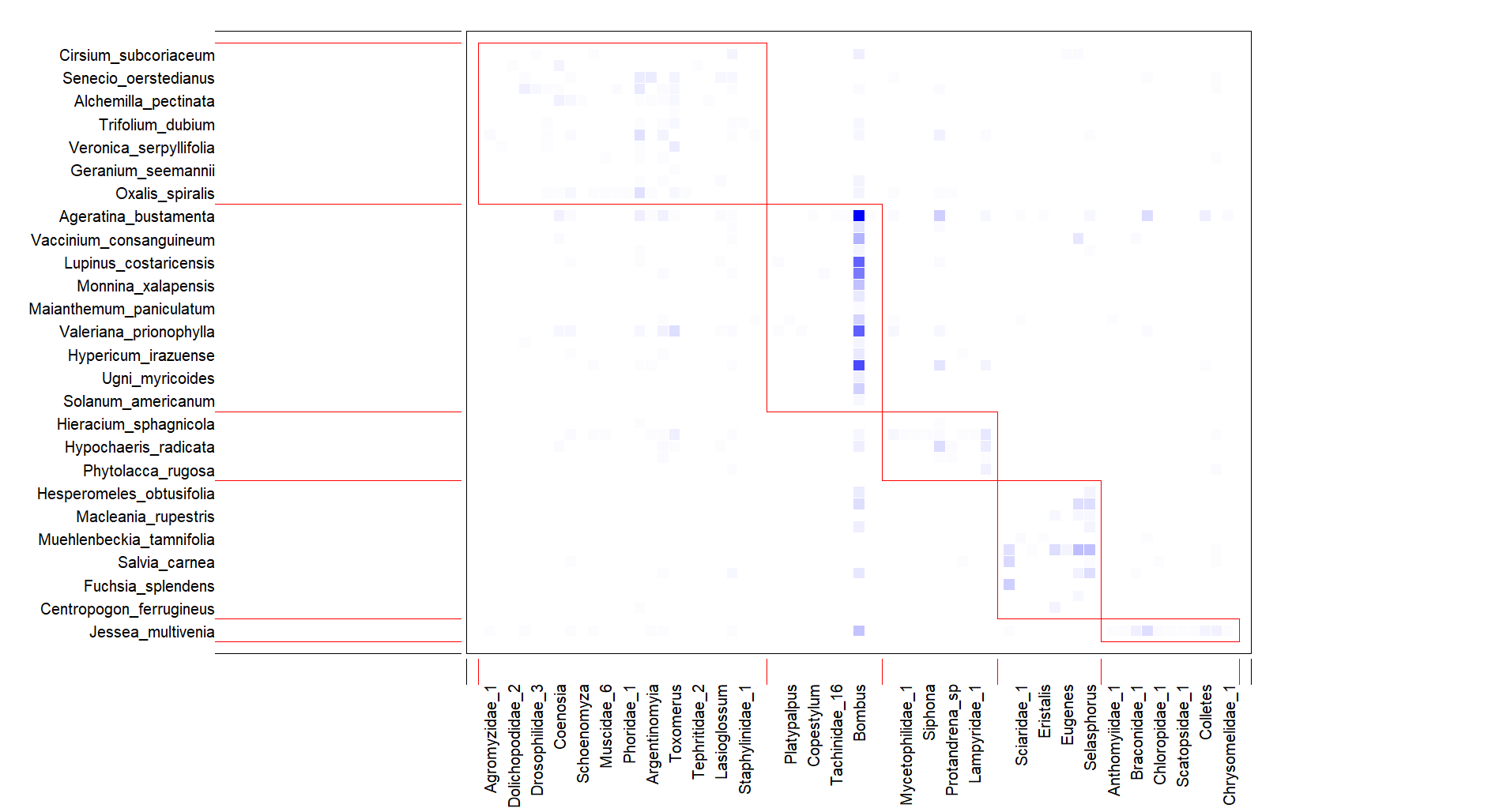


A

C


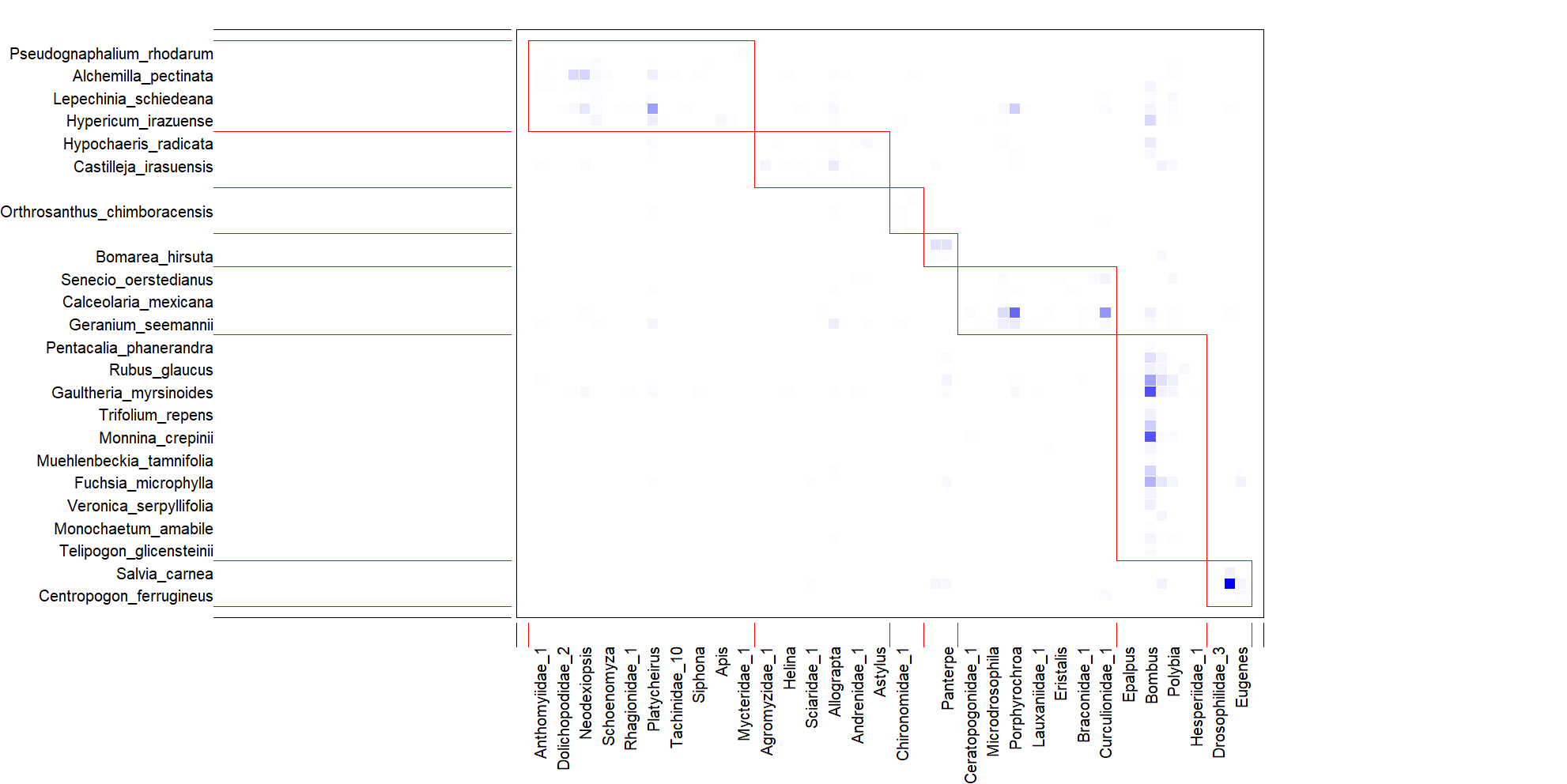


B

D

Fig S2. Modules calculated for each pollination network. A) Montane Forest Dry season, B) Montane Forest Rainy season, C) Paramo Dry season, D) Paramo Rainy season.
