# Supplementary material for: Elevational and seasonal patterns of plant pollinator networks in two highland tropical ecosystems in Costa Rica
Source: PLoS One. 2024 Jan 11;19(1):e0295258. doi: 10.1371/journal.pone.0295258 (PMC10783733; doi:10.1371/journal.pone.0295258)
Supplement: S3 Fig — (DOCX) [file pone.0295258.s003.docx]

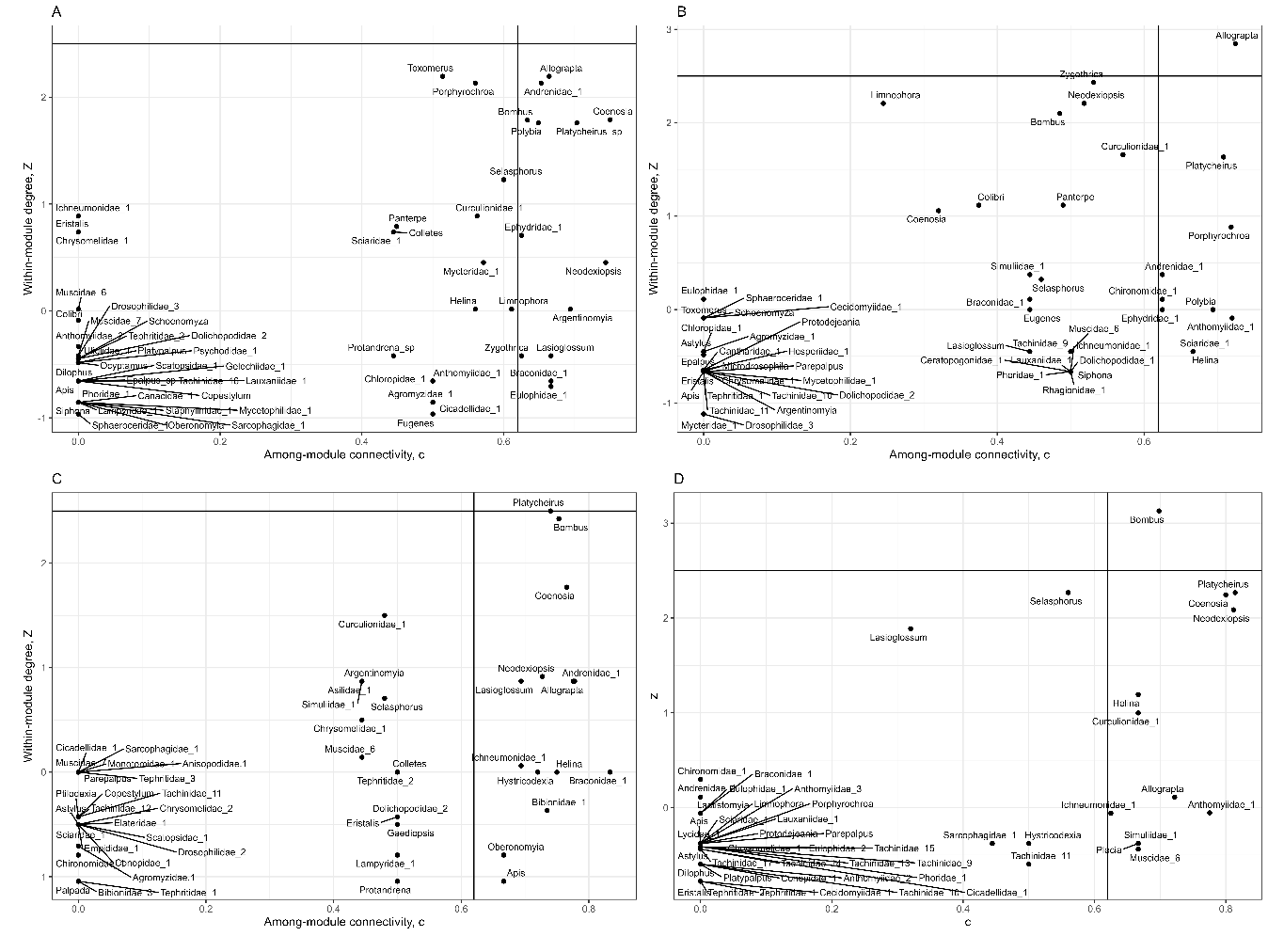


Fig S3. Distribution of floral visitors according to their network role. Each dot represents a species. Lines show the values that separate high and low levels of connectivity as follows: Top left panel: Module hubs; Bottom left panel: peripherals; Top right panel: network hubs; Bottom right panel: connectors (Olesen et al., 2007). a) Montane Forest dry; b) Montane Forest rainy; c) Paramo dry; d) Paramo rainy.
