# Supplementary material for: Elevational and seasonal patterns of plant pollinator networks in two highland tropical ecosystems in Costa Rica
Source: PLoS One. 2024 Jan 11;19(1):e0295258. doi: 10.1371/journal.pone.0295258 (PMC10783733; doi:10.1371/journal.pone.0295258)
Supplement: S2 Table — (DOCX) [file pone.0295258.s005.docx]

S2 Table. Bray-Curtis dissimilarity values of floral visitor communities between ecosystems and seasons

|  | Montane Forest Dry | Montane Forest Rainy | Paramo Dry |
| --- | --- | --- | --- |
| Montane Forest Rainy | 0.39 |  |  |
| Paramo Dry | 0.50 | 0.56 |  |
| Paramo Rainy | 0.65 | 0.58 | 0.48 |
