# Supplementary material for: Elevational and seasonal patterns of plant pollinator networks in two highland tropical ecosystems in Costa Rica
Source: PLoS One. 2024 Jan 11;19(1):e0295258. doi: 10.1371/journal.pone.0295258 (PMC10783733; doi:10.1371/journal.pone.0295258)
Supplement: S3 Table — (DOCX) [file pone.0295258.s006.docx]

S3 Table. Z-scores of each network metric for each season and ecosystem. Z-scores were obtained from the comparison of each empirical matrix with 1000 null matrices generated from the r2d table null model.

|  | **Z-scores** | | | |
| --- | --- | --- | --- | --- |
|  | Paramo | | Montane Forest | |
| **Metrics** | **Rainy** | **Dry** | **Rainy** | **Dry** |
| *H2’* | 38.25 | 28.43 | 70.12 | 59.48 |
| Nestedness (NODF) | -5.20 | -5.06 | -10.21 | -9.85 |
| Weighted nestedness (WNODF) | -4.54 | -5.74 | -9.26 | -7.86 |
| Connectance | -16.68 | -11.02 | -24.36 | -28.26 |
| Interaction evenness | -38.29 | -28.43 | -70.12 | -59.48 |
| Generality (visitors) | -23.89 | -19.73 | -33.02 | -32.50 |
| Vulnerability (plants) | -22.41 | -10.37 | -31.71 | -23.42 |
| Modularity (*Q*) | 34.32 | 23.19 | 61.76 | 52.93 |
